# Synergistic windbreak efficiency of desert vegetation and oasis shelter forests

**DOI:** 10.1371/journal.pone.0312876

**Published:** 2024-10-30

**Authors:** Aishajiang Aili, Xu Hailiang, Abdul Waheed, Fabiola Bakayisire, Xie Yingying

**Affiliations:** State Key Laboratory of Desert and Oasis Ecology, Chinese Academy of Sciences, Xinjiang Institute of Ecology and Geography, Urumqi, China; Feroze Gandhi Degree College, INDIA

## Abstract

This study investigates the novel approach of synergizing desert vegetation with shelter forests to enhance windbreak efficiency in a transitional zone between the Korla oasis and the Taklimakan Desert, northwest China. Through an extensive field survey and experimental setup, we evaluated the impact of different shelterbelt configurations on wind speed reduction. Three types of shelter forests were examined: multi-row Poplar (*Populus alba*), single-row Jujube *(Ziziphus jujube)*, and a mixed-species layout combining one row of Jujube and two rows of Poplar trees. Wind speed measurements were recorded at multiple heights across three zones—open field, between desert vegetation and shelterbelt, and leeward of the shelterbelt—over a three-month period (April to June, 2023). The findings reveal a significant reduction in wind speed, particularly on the leeward side, with multi-row and mixed-species configurations proving the most effective. The highest synergistic efficiency, observed in the mixed-species shelter forest, showed a windbreak efficiency improvement of over 20% compared to desert vegetation alone. This study provides new insights into the combined effectiveness of desert vegetation and shelter forests, offering a strategic framework for designing shelterbelts in arid environments. These results underscore the critical role of diverse, structured vegetation arrangements in combating wind erosion and contribute to the development of sustainable ecological management practices for desert regions worldwide.

## Introduction

Wind erosion is a major concern in arid and semi-arid regions, leading to significant soil degradation, desertification, and biodiversity loss [1]. The establishment of windbreak systems, including natural desert vegetation and artificial shelter forests, is a vital strategy to mitigate the effects of wind erosion by reducing wind speeds and stabilizing soils. While individual contributions of desert vegetation and shelter forests have been well documented, limited research has focused on their synergistic effects, particularly in relation to soil properties such as porosity, which plays a crucial role in windbreak efficiency [2].

Porosity, a measure of the void spaces within soil, directly influences wind erosion processes. High soil porosity can enhance the soil’s ability to retain moisture, reducing wind erosion potential by improving soil cohesion [3]. On the other hand, low porosity soils are more susceptible to wind erosion due to their limited water retention capacity, leading to particle dislodgement by strong winds [4, 5]. Despite the importance of soil porosity in wind erosion dynamics, previous studies have not adequately discussed its interaction with vegetation and shelter forests, which can significantly influence overall windbreak efficiency.

Desert vegetation, characterized by species adapted to harsh, dry environments, plays a critical role in reducing wind velocity near the soil surface, thereby preventing the detachment of soil particles [6]. The natural vegetation helps maintain surface stability and supports increased soil porosity by creating micro-habitats that retain moisture and improve soil structure [7, 8]. These effects are essential in areas where soil is particularly prone to erosion due to high winds and low water availability [9].

Conversely, oasis shelter forests, often established in agricultural and settlement regions, are widely recognized for their ability to form a dense barrier that effectively reduces wind speeds over a wider area [10, 11]. Shelter forests, particularly those in oasis regions, improve windbreak efficiency by altering the wind profile and decreasing wind erosion, while also enhancing the overall porosity of soils through root systems that promote soil aggregation and structure stability [12]. This can result in more stable soils, less susceptible to erosion, even under harsh climatic conditions.

Despite the individual benefits of desert vegetation and shelter forests, their combined influence on windbreak efficiency and soil properties especially porosity remains underexplored [13, 14]. The interaction between these two systems may enhance windbreak efficiency by creating complementary effects on wind speed reduction and soil stabilization. For instance, while desert vegetation may reduce wind speeds near the ground, shelter forests can provide a more substantial reduction at higher elevations, resulting in a multi-layered defense against erosion [15]. Furthermore, the presence of both systems can positively impact soil porosity by improving moisture retention and supporting microbial activity, leading to greater soil cohesion and reduced erosion risk.

Located at the interface of the Korla oasis and the Taklimakan desert, the study area is characterized by unique climatic and geographical conditions that exacerbate wind erosion [16]. By integrating field measurements and digital image analysis, this research affirms that well-designed shelter forests are crucial in enhancing the natural defenses of desert environments, offering significant benefits in terms of wind speed reduction. The study provides a comprehensive evaluation of vegetation structures and their roles in controlling wind speed, with practical implications for environmental management and sustainable land use in desert regions. In particular, it assesses the synergistic effects of desert vegetation and oasis shelter forests on windbreak efficiency, with a focus on soil porosity. The research quantifies wind speed reduction and analyzes soil characteristics under different vegetation configurations, offering insights into optimizing land management practices to combat desertification. Additionally, the role of porosity in improving soil resilience and stability is explored, contributing to a deeper understanding of how desert-oasis ecosystems can be better managed for sustainable development. The findings of this study will be valuable for policymakers and land managers in designing effective strategies to promote ecological stability in vulnerable landscapes.

This study aims to assess the synergistic effects of desert vegetation and oasis shelter forests on windbreak efficiency, with a particular focus on soil porosity. By quantifying wind speed reduction and analyzing soil characteristics under different vegetation configurations, we seek to provide insights into optimizing land management practices to mitigate desertification. Additionally, the role of porosity in improving soil resilience and stability will be explored, contributing to a deeper understanding of how desert-oasis ecosystems can be better managed for sustainable development.

## Data and method

### Site selection

**Fig 1:**
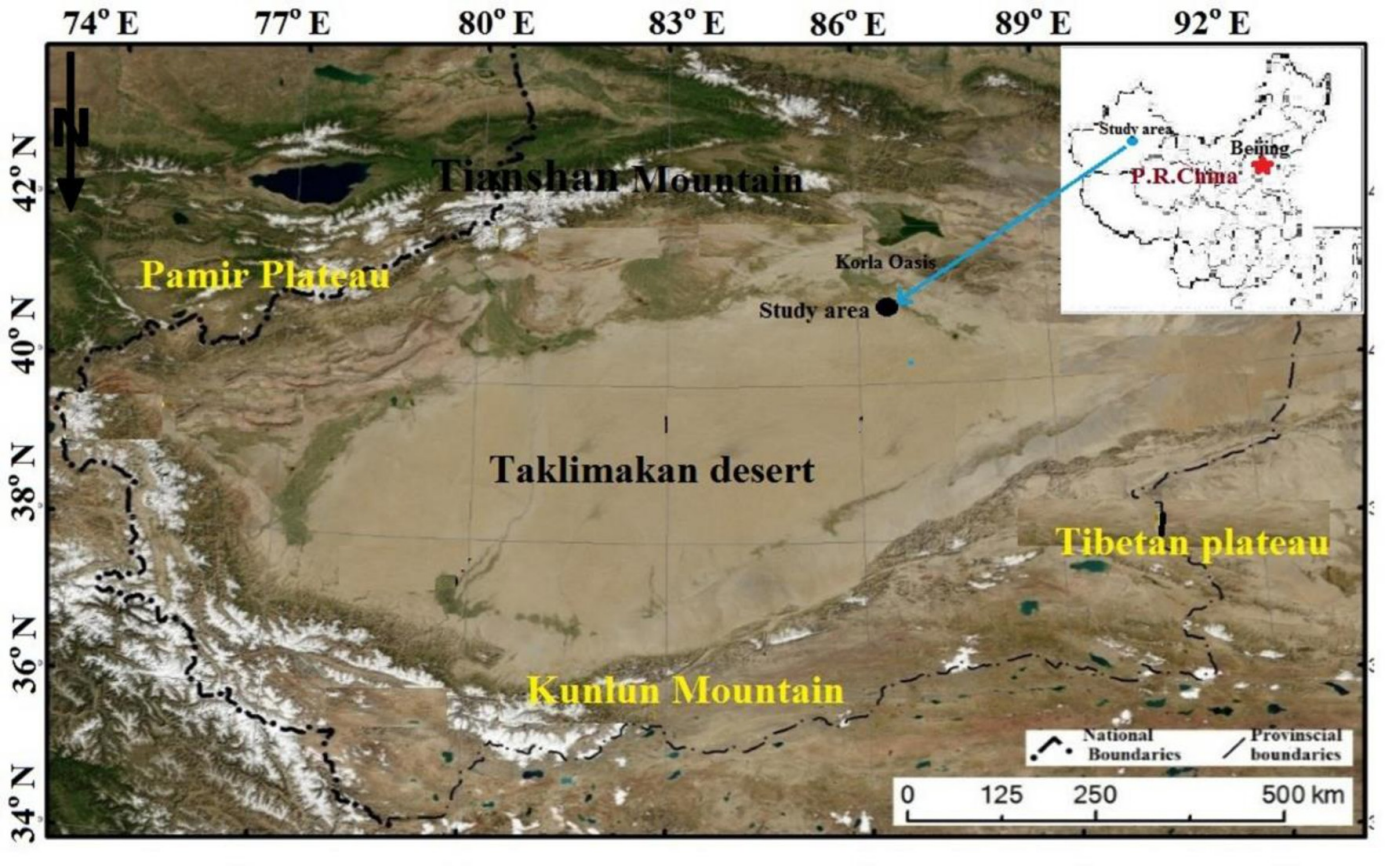
Location and surrounding environments of the study area (driving number of map: GS (2019)1822).

The study was conducted in the transitional zone between the Korla Oasis and the Taklimakan Desert, located in northwest China’s Xinjiang Uygur Autonomous Region (Fig 1). This area lies between the Taklimakan Desert to the south and the Kurukkum Desert to the northwest, and is strategically positioned between these vast desert systems. Shielded by the Tianshan Mountains to the north and east, the region intercepts cold winds and moist air from the Yanji Basin, which intensifies its arid conditions. The area experiences a warm temperate continental dry climate, with an annual average temperature of 11.4°C, 2990 hours of sunshine, and a frost-free period of 210 days annually. Precipitation is scarce, with an annual average of 58.6 mm, while evaporation is extremely high, reaching 2788.2 mm, contributing to severe water scarcity in the region [17, 18]. The complex local atmospheric circulation, combined with frequent sandy dust weather events, makes this region a natural laboratory for studying windbreak effectiveness. Given these challenging conditions, the study site is ideal for exploring the synergistic effects of desert vegetation and shelterbelt systems on wind speed reduction. The unique environmental conditions provide important insights into wind erosion control strategies that can be applied in similar arid regions globally [19–21].

### Collection of field investigation data

#### Investigation of shelterbelt structure

As for the investigation of shelterbelt structure, this study adopted the field investigation method, that is, to set up a 50m of long temporary standard land, and then investigate the shelterbelts within the standard land by each tree and the forest belt composition. Total of 12 shelter forest belt were selected by using above method. Among them, shelterbelt 1, 2, 3 and 4 were composed of three rows of poplar trees (*Populus alba*); Shelterbelt 5,6,7 and 8 were composed of one row of jujube trees *(Ziziphus jujube)*, while the shelterbelt 9,10,11 and 12 were composed of one row of jujube trees and two rows of poplar trees. The investigation indexes were: tree species composition, belt configuration, row spacing, row number, belt width, Diameter at Breast Height (DBH), crown width, average tree height, and average height under branch and the average crown height. The porosity of shelterbelt were measured by using pixel method.

#### Measurement of shelterbelt porosity

In this study, image processing software was used to measure the porosity of shelterbelt [22, 23]. Firstly, the camera was used to take photos of the shelter forest, the photo number and the location of the shelter forest were recorded, and the obtained images were brought back to the laboratory and imported into the image processing software Photoshop CS5 for preprocessing, mainly for image cropping and brightness adjustment of the obtained shelter forest images, and the images were divided into two parts: tree crown and tree trunk. The gray threshold method is used to distinguish the shelter forest part from the light transmittance part in the shelter forest image, and then the pixel proportion of the light transmittance part in the image is calculated respectively, where the pixel proportion of the light transmittance part is the two-dimensional thickness of the forest belt. The two-dimensional permeability of shelterbelt is calculated using the following formula:

$$\beta =(\beta_{1}\times {\text{h}}_{1}+\beta_{2}+{\text{h}}_{2})/\text{H}$$

$$\beta^{1}=(\beta_{3}\times {\text{n}}_{1}+\beta_{4}\times {\text{n}}_{2})/\left({\text{n}}_{1}+{\text{n}}_{2}\right)$$ (Formula 1)

where, β and β1 represent two-dimensional porosity of single species shelterbelt and mixed forest belt; H, h1 and h2 represent mean height of forest belt, mean crown height and under branch height; β1 and β2 represent canopy porosity and trunk porosity; β3 and β4 represent windward and leeward porosity; n1 and n2 are the retention rates of tree species 1 and tree species 2, respectively.

#### Measurement of wind speed

**Fig 2:**
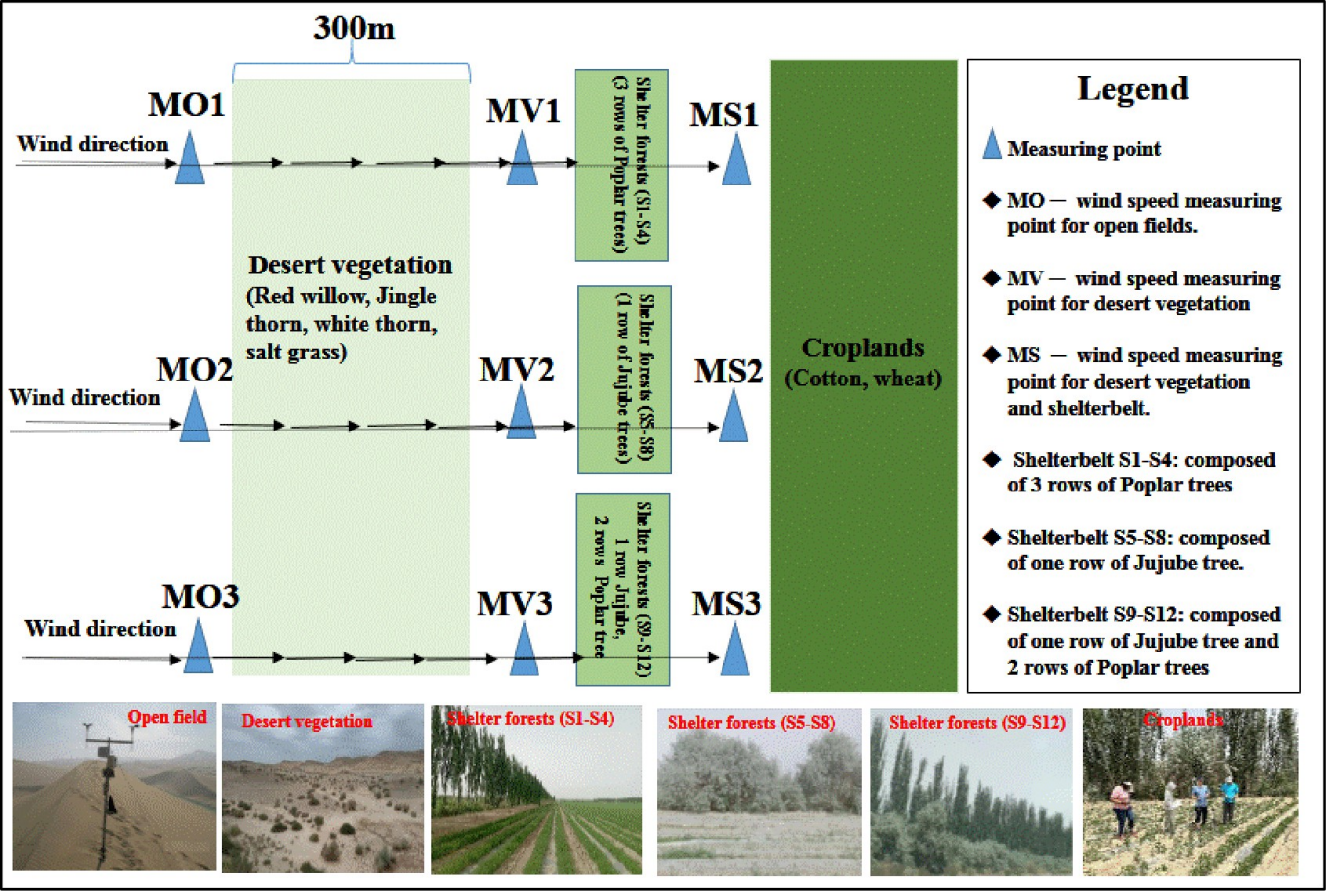
Distribution of wind speed monitoring point.

To assess the combined windbreak efficiency of natural vegetation and shelterbelts, both were evaluated as an integrated system rather than individually. From April to June 2023, a total of 38 days with wind direction perpendicular to the shelter forests were selected as the detection of the windbreak efficiency of the shelterbelt. In this study, wind speed observation points were strategically placed to assess the synergistic windbreak efficiency of shelter forests and desert vegetation. The measuring point inside the shelterbelt (MS) was set up to capture wind speed reduction within the forest. Additional points were positioned at 5m, 15m, and 30m behind the shelterbelt on the leeward side to track wind speed decreases as it moved through and beyond the forest. A baseline measurement point (MO) was placed 300m from the shelterbelt in the open field to measure wind speeds without obstruction. These placements ensured accurate assessment of wind reduction patterns, providing clear insights into the combined windbreak efficiency of the vegetation and shelter forests. Firstly, a measuring point (MO) was established 300 meters away from the shelter forest, in the direction of the oasis, and was used primarily to measure wind speed in the open field. Secondly, the transition point (MV) was set up, which was located between the shelter forest and the natural vegetation, and the windbreak efficiency of the shelter forest could be analyzed by comparing the wind speed of MO and MV. Thirdly, a measuring point (MS) is set up inside the shelter forests, and the synergistic windbreak efficiency of natural vegetation and shelter forest could be obtained by comparing the wind speed of MS and MV. In the field observation process, in order to reduce the measurement error caused by the difference of underlying surface, two AVM-03 wind speed/temperature meters were installed at each measuring point at three heights (0.5m,1.2m, and 2.0m), and the wind speed were measured at the same time. Distribution of the monitoring point are shown in the Fig 2 (Fig 2). The results of the three wind speed were averaged as for each of measuring point data.

#### Calculation of relative wind speed and windbreak efficiency

Relative wind speed is often used to measure the windbreak efficiency. In this study, relative wind speed is used as an index for measuring the windbreak efficiency of natural vegetation and shelter forest following the method of “Reference 24” [24]. The specific calculation formula is as follows:

$$\text{RV}\%=\text{U}/{\text{U}}_{0}*100$$ (Formula 2)

Where, RV is the relative wind speed, U is the wind speed value of the leeward side of the shelter forest belt, U_0_ is the wind speed of the control point at the same time, and the windbreak efficiency is (100-RV) %.

## Results

### Porosity of shelterbelt

**Fig 3:**
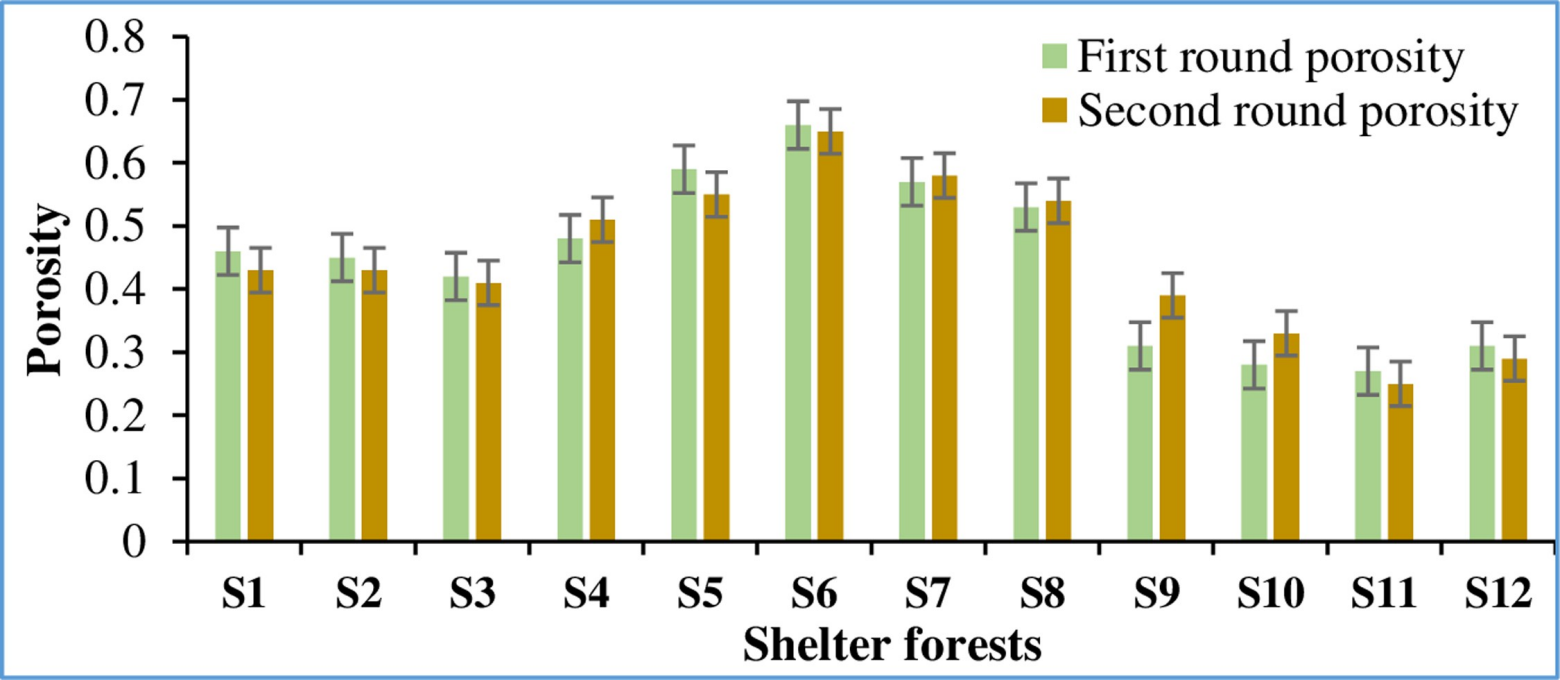
Porosity of different types of shelterbelt.

Due to the projection error and image processing error in the digital image processing method used in the study, the obtained results of the shelter forest porosity were analyzed to test the influence of the above errors on the experimental results. The results of the obtained 12 forest belts were divided into two groups for one-way ANOVA. Porosity of selected shelter forests were presented in the Fig 3.

As shown in Fig 3, the porosity of the different shelter forest types is categorized into three distinct groups. It can be seen from the Fig 3 that, composition and the height of the shelter forests determines its porosity. First type of Shelter forests (SI-S4), consisting of multiple rows of a single type of tree, specifically three rows of Poplar trees, shows porosity values of 0.43, 0.43, 0.41, and 0.51 respectively, with an average porosity of 0.45.

Second type of Shelter forests (S5-S8), consisting of a single row of a single type of tree, in this case, Jujube trees, has higher porosity values ranging from 0.54 to 0.65, averaging at 0.58. Third type of Shelter forests (S9-S12), a mixed composition featuring multiple trees with multiple rows, including one row of Jujube trees and two rows of Poplar trees, records the lowest porosity figures, from 0.25 to 0.39, averaging at 0.30. The data indicates that the second type of shelter forest (single row of Jujube trees) exhibits higher porosity compared to the others. This suggests that less dense configurations may allow more space for air and moisture to permeate, which is characteristic of higher porosity. The third type, which combines multiple species and rows, shows significantly lower porosity. This might be attributed to the denser arrangement and potentially overlapping canopy layers reducing the space for air. The first type, while being multi-row, is homogeneous in species and shows moderate porosity. The consistency in species might contribute to a more uniform growth pattern that does not drastically hinder or promote porosity.

### Variation of wind speed under the influence of desert vegetation and different Shelterbelt

**Fig 4:**
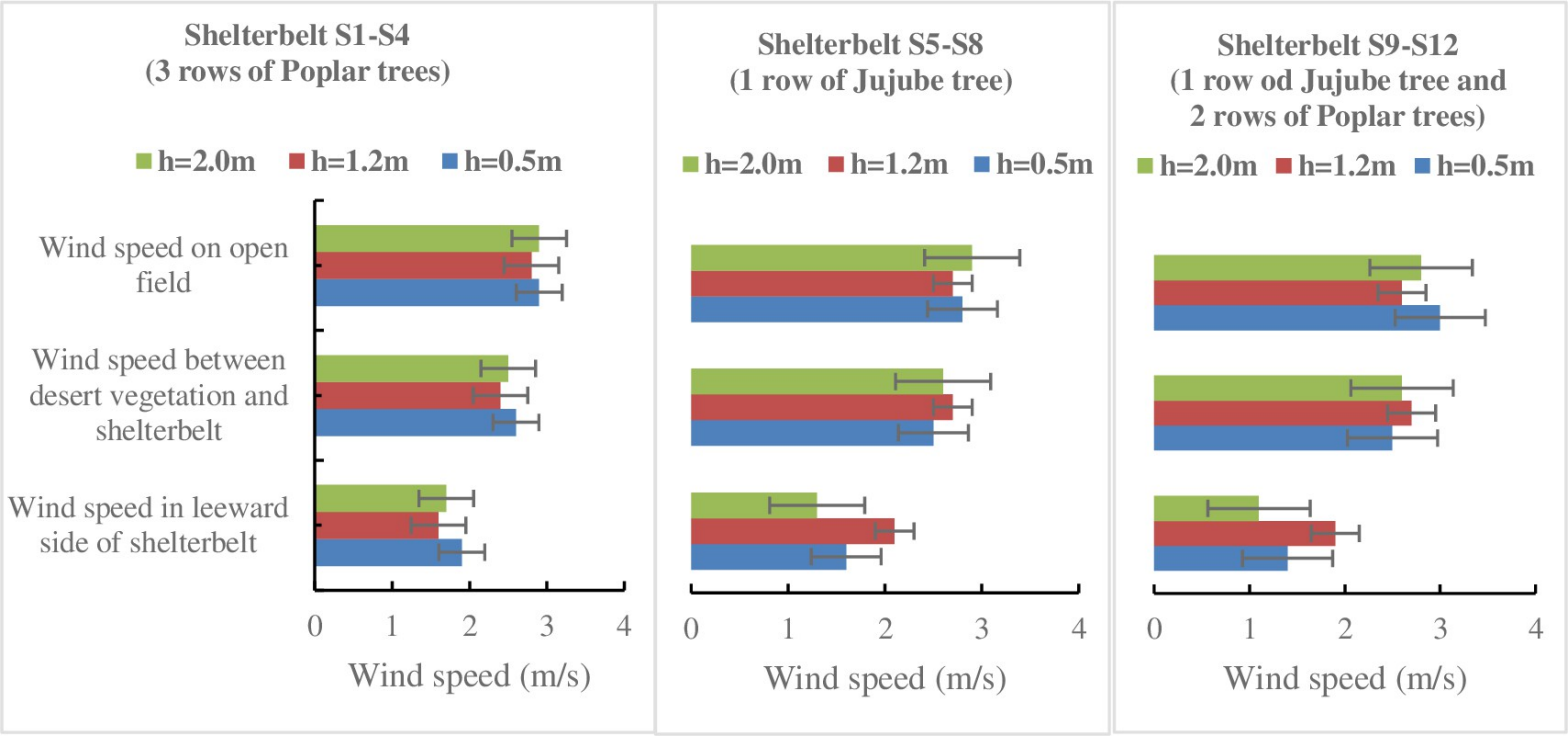
Wind speed under the influence of desert vegetation and different shelterbelt. The error bars represent standard deviations (SD).

Fig 4 shows the wind speeds measured at different heights (0.5m, 1.2m, and 2.0m) across various fields influenced by desert vegetation and three types of shelter forests. The forests vary in structure and species: Shelter type 1 (S1-S4) consists of three rows of Poplar trees; Shelter type 2 (S5-S8) comprises a single row of Jujube trees; and Shelter type 3 (S9- S12) includes one row of Jujube trees with two rows of Poplar trees. Measurements were taken in open fields, between desert vegetation and shelterbelts, and on the leeward side of the shelterbelt.

The results presented in Fig 4 reveals that the presence of shelter forests significantly influences wind speeds, especially on the leeward side, where wind speed reduction is most pronounced. For instance, in the first type of shelter forest, the leeward side wind speeds range from 1.9 m/s at 0.5m height to 1.7 m/s at 2.0m, compared to open field speeds of approximately 2.9 m/s. This trend of reduction holds across all forest types and heights, indicating effective wind speed mitigation by the shelterbelt structures. Discussion of the data shows that the multi-row configurations in the first and third types of forests are more effective in reducing wind speeds than the single-row configuration in the second type. For example, at a 0.5m height, wind speeds on the leeward side are reduced to 1.9 m/s and 1.4 m/s in the first and third types respectively, compared to 1.6 m/s in the second type. This suggests that the density and complexity of the forest structure play crucial roles in its effectiveness as a windbreak. Further analysis reveals that wind speeds within the sheltered areas (between the desert vegetation and the shelterbelt) are consistently lower than the wind speed observed in the open field, yet slightly higher than on the leeward side, indicating some penetration and slowdown of wind through the shelterbelt before further reduction on the leeward side. This gradient of wind speed from open fields through the sheltered area to the leeward side showcases the progressive impact of shelterbelts in managing wind flow, with maximum reduction occurring as wind passes completely through the shelterbelt.

Variation of wind speed on different fields underscores the effectiveness of shelter forests in reducing wind speeds, particularly on the leeward side, with denser and multi-row forests providing more substantial protection against wind. This highlights the importance of strategic shelter forest design in mitigating wind impact in susceptible areas, potentially contributing to better soil stability and reduced erosion in desert environments.

### Synergistic windbreak efficiency of desert vegetation and shelter forest

**Fig 5:**
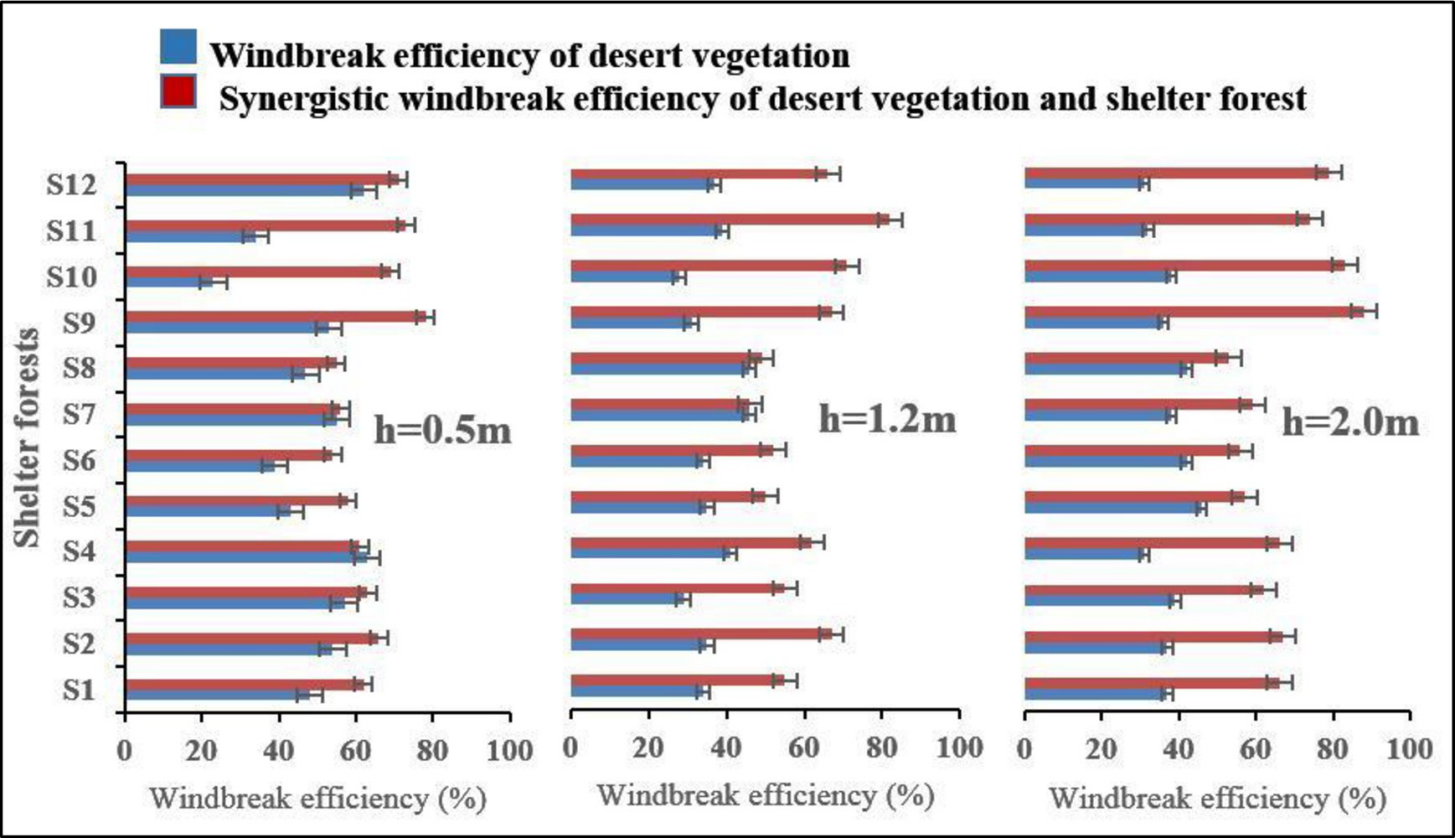
Synergistic windbreak efficiency of desert vegetation and shelter forest. The error bars represent standard deviations (SD).

Fig 5 presents a comparative analysis of windbreak efficiency between desert vegetation alone and the synergistic effect when combined with different types of shelter forests at three different heights (0.5m, 1.2m, and 2.0m).

In examining the synergistic windbreak efficiency within different forest types, it is clear that each forest configuration yields distinct results. The first type, with multiple rows of Poplars, typically enhances windbreak efficiency more effectively as the height increases, showing notable improvements over the efficiency provided by desert vegetation alone. For instance, at 2.0 meters, the synergistic efficiency increases to 58% from a lower base of 36% offered by desert vegetation in S4. This suggests that denser, layered forest structures are better at disrupting wind flow at higher elevations. The data reveals that the synergistic windbreak efficiency, which measures the combined effect of desert vegetation and shelter forests, is consistently higher across all types and heights compared to the windbreak efficiency of desert vegetation alone. For example, at the 0.5m height, the synergistic efficiency in the first shelter forest type ranges from 59% to 67%, significantly higher than the 48% to 63% provided by the desert vegetation alone. Comparatively, the second type, featuring a single row of Jujube trees, provides a significant increase in efficiency at lower heights, demonstrating that even a singular row can be effective given the right species. In trees with taller trunks and branches beginning higher above the ground, the wind reduction near the ground may be less effective compared to trees with shorter trunks and branches originating closer to the surface, where wind reduction is greatest near the ground level. However, the impact tends to diminish as the height increases, as seen in the lesser improvement at 2.0 meters. On the other hand, the third type, which combines both Jujube and Poplar trees in a multi-layered configuration, shows the highest synergistic efficiency, particularly at 2.0 meters with an efficiency of 88% in S12. This configuration provides an excellent example of how combining different species and multiple layers can create the most effective windbreak, confirming the superior performance of diverse, structured shelter forests over single-species, single-row configurations.

This trend continues at higher measuring points, with the synergistic efficiency often exceeding the solo vegetation efficiency by more than 20% in some cases. This analysis underscores the enhanced protection against wind erosion provided by integrating shelter forests with desert vegetation. The third type of shelter forest, which features a mix of Jujube and Poplar trees, generally shows the highest synergistic windbreak efficiency, particularly at a height of 2.0m, where it reaches up to 88%. This suggests that a diverse and multi-layered forest structure might be more effective at disrupting wind flow and reducing wind speed compared to simpler, single-row configurations. These findings illustrate the critical role of combining different types of vegetation to optimize windbreak efficiency. They provide vital insights for ecological and landscape management, demonstrating that well-planned shelter forest arrangements can significantly bolster the natural defense mechanisms of desert vegetation against adverse environmental conditions.

## Discussion

The synergistic effect of desert vegetation and shelter forests on wind speed reduction, as demonstrated in this study, provides valuable insights for the design and optimization of windbreak systems in arid regions. The results show that combining natural desert vegetation with shelterbelts significantly enhances windbreak efficiency, particularly in multi-row and mixed-species configurations. This enhanced efficiency, with over 20% greater reduction in wind speed compared to desert vegetation alone, underscores the importance of diversity and structural complexity in shelterbelt design. By disrupting wind flow more effectively, these configurations offer stronger protection against wind erosion, making them a practical solution for mitigating desertification. The findings suggest that integrating both natural and artificial vegetation in landscape planning can play a critical role in maintaining ecosystem stability, reducing soil erosion, and safeguarding agricultural productivity in harsh desert environments.

### Influence of shelterbelt structure and porosity on wind speed reduction

The results from this study highlight the critical role that shelterbelt structure and porosity play in determining the effectiveness of windbreaks. Shelter forests with different configurations and species compositions were found to vary in their porosity, which in turn influences their capacity to reduce wind speeds. Forests composed of multiple rows of poplar trees (Shelterbelt types S1-S4) demonstrated moderate porosity and significant wind speed reduction. This suggests that denser tree configurations might be more effective at blocking wind, particularly at lower heights.

In contrast, single-row configurations of jujube trees (Shelterbelt types S5-S8) exhibited higher porosity but slightly less effectiveness in wind speed reduction compared to multi-row configurations. This indicates that while higher porosity allows for some wind penetration, it might not be as effective at significantly reducing wind speed unless combined with other rows or types of trees. Single-row windbreaks, although more economical in terms of land and resource use, may provide limited wind reduction compared to multi-row windbreaks, particularly at lower heights where wind speeds are crucial for preventing soil erosion. Research has shown that single-row windbreaks can reduce wind speeds but may allow more wind to pass through at higher altitudes, depending on the porosity and structure of the vegetation [25] In contrast, multi-row windbreaks offer a more robust defense by creating a denser barrier, which slows wind speeds more effectively at various heights, thereby providing greater overall protection. This has been supported by several studies showing that multi-row configurations, especially those with a mix of vegetation species and heights, tend to be more effective in reducing wind speed over longer distances [26, 27].The mixed species shelterbelts (S9-S12), combining both jujube and poplar trees, showed the lowest porosity and the highest effectiveness in reducing wind speeds at all measured heights. This suggests that a combination of different tree species and configurations can synergistically enhance the shelterbelt’s ability to disrupt and reduce wind flow. Our findings were consistent with previous research results. Previously, a study found that, an optimal range of 20–40% optical porosity as turbulence created by very dense shelterbelts reduces the extent of the sheltered area. Within this range, the magnitude of wind speed reduction generally increases with decreasing optical porosity [28] Based on optical porosity alone, the pine and mixed native compositions of shelter forests will provide a greater magnitude of wind speed reduction compared to eucalypt shelterbelts [29, 30].

### Synergistic effects of desert vegetation and shelter forests

The integration of natural desert vegetation with structured shelterbelts demonstrates a clear synergistic effect on wind speed reduction. The comparative analysis between areas with only desert vegetation and those with both vegetation and shelterbelts shows that the combined approach significantly enhances windbreak efficiency. This synergistic effect is most pronounced in the mixed-species, multi-row shelterbelts, where the diversity in tree species and structural complexity likely creates a more variable and turbulent flow that further reduces wind speed. Bird et al. [31] conducted similar research and confirmed that, despite pine shelterbelts having low overall porosity and greater height, an increase in porosity at the lower strata can reduce wind speed mitigation to such an extent that it offsets the benefits typically associated with shelter distance

The presence of natural desert vegetation alone provides some degree of wind speed reduction, which is notably less than that achieved when combined with shelterbelts. This finding underscores the importance of integrating multiple types of vegetation and structural strategies in the design of effective windbreak systems in desert environments. The increased protection against wind erosion, particularly at greater heights, highlights the potential for these combined systems to significantly impact soil stability and reduce erosion over time.

### Implications for shelterbelt design and management

The insights gained from this study have important implications for the design and management of shelterbelts in arid and semi-arid regions. The effectiveness of shelterbelts in reducing wind speed, particularly on the leeward side, suggests that strategic placement and configuration of these structures could enhance their utility in environmental management and land-use planning. The synergistic windbreak efficiency of natural forests and shelterbelt were also confirmed in previous research [32–36]. Additionally, the significant role of shelterbelt porosity in influencing wind speed reduction offers a valuable parameter for optimizing the design of future windbreaks to maximize their ecological and protective benefits.

### Practical implications and environmental impact

The findings of this study suggest that the combined use of desert vegetation and shelter forests offers superior windbreak efficiency compared to traditional single-layered windbreak approaches. By integrating diverse vegetation types and multi-row shelterbelt designs, the synergistic effect enhances wind speed reduction, resulting in a more effective barrier against wind erosion. This approach provides a sustainable alternative to conventional methods, which often rely on single-species or single-row shelterbelts that may not offer the same level of protection in harsh desert conditions.

The improved windbreak efficiency observed in this study, especially in the mixed-species and multi-row configurations, can be directly applied to arid and semi-arid regions facing similar environmental challenges. These findings offer practical guidance for landscape management and ecological restoration efforts, emphasizing the importance of biodiversity and structural complexity in shelterbelt design. Implementing these strategies could play a significant role in mitigating wind erosion, preserving soil quality, and enhancing the resilience of agricultural and ecological systems in desert regions globally.

Overall, the interaction between desert vegetation and shelter forests presents a compelling strategy for managing wind flow and reducing erosion in vulnerable desert landscapes. The data from this study not only validate the effectiveness of existing shelterbelt configurations but also provide a foundation for exploring innovative designs that leverage the unique characteristics of both natural and man-made vegetative structures. The optimization of shelterbelt design based on structural and species diversity could lead to more robust environmental protection strategies that are critically needed in the face of escalating climate challenges in arid regions.

## Conclusion

This study offers a comprehensive evaluation of the synergistic windbreak efficiencies of desert vegetation combined with various shelter forest configurations in the transitional zone between the Korla oasis and the Taklimakan desert. Through rigorous field measurements and image processing analyses, the findings highlight the critical role of shelter forests in mitigating wind speeds and enhancing the stability of desert environments against wind erosion. The results confirm that shelter forests, particularly those with three-row configurations—whether composed solely of Poplar trees or a mix of Poplar and Jujube trees—outperform single-row configurations in wind speed reduction. The increased density and complexity of these forests significantly improve their windbreak efficiency, with the mixed-species configuration achieving optimal porosity (∼0.30) and the highest windbreak effectiveness.

The most notable achievement of this research is the demonstration of the synergistic effect of combining desert vegetation with shelter forests, which increased windbreak efficiency by more than 20% compared to desert vegetation alone. This suggests that integrating diverse vegetation types into shelterbelt designs can substantially enhance their protective function against wind erosion. The implications of these findings are profound for ecological management and landscape planning in arid and semi-arid regions. Strategic design and placement of shelter forests offer a cost-effective and environmentally sustainable approach to stabilizing ecosystems, reducing soil erosion, and protecting agricultural lands from adverse climatic conditions.

Future research should focus on the long-term impacts of these shelterbelt configurations, particularly in terms of their resilience to climate variability and their broader ecological effects, including biodiversity and soil health. Additionally, further studies could explore optimal shelterbelt configurations for different environmental conditions and terrains, providing more tailored solutions for regions facing wind erosion and related challenges.
